# Rail Mounted Gantry Crane Scheduling Optimization in Railway Container Terminal Based on Hybrid Handling Mode

**DOI:** 10.1155/2014/682486

**Published:** 2014-11-04

**Authors:** Li Wang, Xiaoning Zhu

**Affiliations:** School of Traffic and Transportation, Beijing Jiaotong University, Beijing 100044, China

## Abstract

Rail mounted gantry crane (RMGC) scheduling is important in reducing makespan of handling operation and improving container handling efficiency. In this paper, we present an RMGC scheduling optimization model, whose objective is to determine an optimization handling sequence in order to minimize RMGC idle load time in handling tasks. An ant colony optimization is proposed to obtain near optimal solutions. Computational experiments on a specific railway container terminal are conducted to illustrate the proposed model and solution algorithm. The results show that the proposed method is effective in reducing the idle load time of RMGC.

## 1. Introduction

Container transportation is an advanced transportation mode and plays an important role in international freight transportation. As an important form of container transportation organization, railway container transportation integrates the advantages of container and railway transport and has characteristics of safety, convenience, energy saving, environmental protection, and door to door transport. In railway container transportation systems, container trains move massive quantities of containers over long distances, and trucks are used for short distance pick-up and delivery activities. To ensure rapid container transfer between rail and truck, modern railway container terminals are required, where they have advanced equipment, establishments, and efficient management strategies including organizing, scheduling, operating, and so forth. The productivity of railway container terminal has a significant impact on transportation quality, comprehensive efficiency, and service level of railway transportation network and multimodal transportation system.

Since 2006, 18 modern railway container terminals have been planned and constructed in China, which have advanced arrival-departure lines, storage space, and handling equipment (RMGC, reach stacker, etc.). But the current scheduling method in railway container terminal cannot meet the developing demands of container transportation in China. So it is necessary for railway container terminals to optimize resources utilization.

As a key resource in railway container terminals, RMGC is responsible for containers handling and stockpiling in main operation area. An RMGC scheduling specifies the handling sequence of containers among trains, trucks, and blocks and the time schedule for handling tasks. The RMGC scheduling is a vital part of resources utilization in railway container terminals.

In this paper, we formulate and solve the RMGC scheduling problem in railway container terminal under hybrid handling mode. The rest of paper is organized as follows. The relevant literature is reviewed in Section 2. The RMGC scheduling problem is described in Section 3 and formulated in Section 4. An ant colony optimization is developed in Section 5. Computational results are reported in Section 6 and finally Section 7 covers the conclusion.

## 2. Literature Review

RMGC scheduling problem of railway container terminal belongs to the crane scheduling problem (CSP) which is defined as allocating cranes to handle the loading-unloading operations according to the handling modes and rules with the aim of determining optimization handling sequence in order to minimize the makespan or total completion time of handling task.

According to the different kinds of terminals, CSP can be divided into CSP in marine container terminals and CSP in railway container terminals. The CSP in marine container terminals is the hotspot of CSP research and can be classified into quay crane scheduling problem (QCSP) and yard crane scheduling problem (YCSP).

Daganzo first discussed the QCSP in 1989 and presented exact and approximate solution methods for determining the number of cranes to assign to ship bays of multiple vessels [1]. Based on the study of Daganzo, Peterkofsky and Daganzo proposed a branch and bound method for practical quay crane scheduling problem. However, the above studies did not consider the interference among QCs or precedence relationships among tasks [2]. Kim and Park further investigated QCSP by considering various interference possibilities between adjacent cranes and proposed a mixed integer programming model to determine starting and ending times for each quay crane to serve each ship bay [3]. Ng and Mak considered the QCSP and proposed a heuristic algorithm, which first decomposes the difficult multicrane scheduling problem into easier subproblems by partitioning the ship into a set of nonoverlapping zones [4]. Lee et al. proved the QCSP with noninterference constraints is NP-complete and provided a more concise mathematical model of QCSP [5]. Unsal and Oguz proposed a constraint programming (CP) model for QCSP, which considers realistic constraints such as safety margins, travel times, and precedence relations [6]. Chen et al. present a more compact mathematical formulation of the unidirectional cluster-based QCSP that can be easily solved by a standard optimization solver [7].

Hwan Kim and Bae Kim considered the routing transfer cranes problem of container yard during loading operations of export containers at marine terminals. A mixed integer program model was proposed to minimize the total container handling time of a transfer crane, which includes setup time at each yard bay and travel time between yard bays [8]. Ng and Mak investigated YCSP to schedule a yard crane for a given set of loading/unloading jobs with different ready times. The objective is to minimize the sum of job waiting times and a branch and bound algorithm is proposed to solve the scheduling problem optimally [9]. Li et al. develop an efficient model for YCSP by taking into account realistic operational constraints such as intercrane interference, fixed YC separation distances, and simultaneous container storage/retrievals [10]. Chang et al. present a novel dynamic rolling-horizon decision strategy to solve YCSP and proposed an integer programming model to minimize the total task delaying at blocks [11]. Lee et al. considered the integrated problem for bay allocation and yard crane scheduling in transshipment container terminals. A mixed integer programming model was proposed with the objective of minimizing total costs, including yard crane cost and delay cost [12]. Gharehgozli et al. formulated YCSP as an integer model, proved the problem complexity, and developed a two-phase solution method to obtain optimal solutions [13].

According to the literature retrieval of crane scheduling problem, we can observe that current research specifically focuses on CSP in marine container terminals. The studies on QCSP and YCSP have been conducted by various researchers, not merely limited to the literatures mentioned above. By contrast, specific literature on CSP in railway container terminal is scare. The different operation procedure and rules of cranes between railway and marine container terminals lead relevant research achievements of QCSP and YCSP cannot be directly applied in railway container terminals. Boysen and Fliedner and Boysen et al. divided CSP in railway container terminals into two parts, including assigning container moves to RMGCs and deciding on the sequence of container moves per-RMGC [14, 15]. Their studies focused on the first part to study the crane scheduling problem with fixed crane areas in rail-truck and rail-rail transshipment yards. In this paper, we consider the RMGC scheduling problem in railway container terminals. Our study focuses on the second part to determine optimization sequence of container moves per-RMGC in order to minimize RMGC idle load time in handling tasks.

## 3. Problem Description

The handling area, objects, mode, and scheduling objective of RMGC scheduling problem in railway container terminals are described in this section.

### 3.1. Handling Area of RMGC

**Figure 1:**
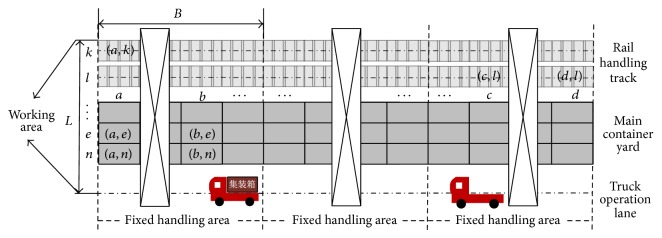
Handling area of per-RMGC.

Based on the length of rail handling track and RMGC amount, the operation area can be equally divided and each RMGC is responsible for one fixed handling area. A dividing instance is shown in Figure 1. This dividing mode can well balance the utilization of RMGCs, avoid intercrane interference, and is used in most of railway container terminals in China. Therefore, our study is based on this mode.

### 3.2. Handling Objects of RMGC

**Figure 2:**
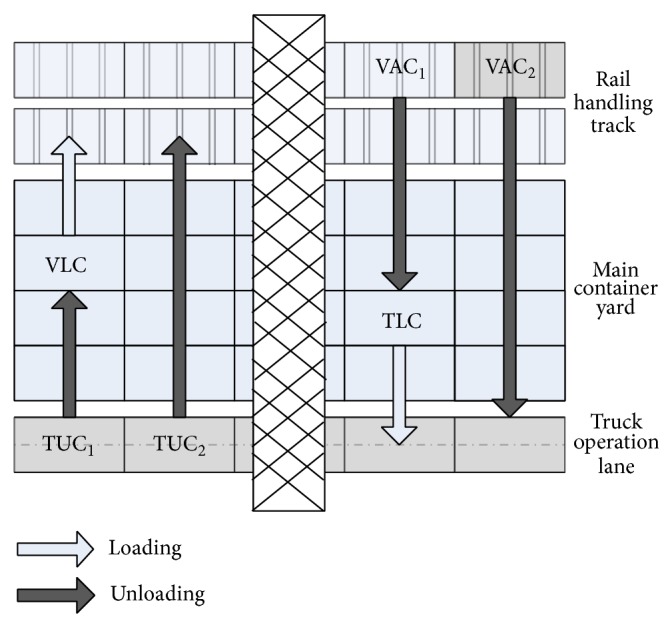
Handling operations of four-type containers.

According to the different handling stage, containers in railway container terminals can be classified into the following four types. The handling operations of four-type containers are shown in Figure 2.

1. Vehicle unloading containers (VAC): inbound containers on rail vehicles before they are unloaded. VAC_1_ are allocated to container yard and VAC_2_ are directly unloaded to trucks.
2. Truck unloading containers (TUC): outbound containers brought in terminal by trucks. TUC_1_ are allocated in container yard and TUC_2_ are directly unloaded to vehicles.
3. Vehicle loading containers (VLC): outbound containers already in container yard waiting for loading to rail vehicles.
4. Truck loading containers (TLC): inbound containers already in container yard waiting for loading to trucks to customers.

### 3.3. Handling Mode

The handling mode of cranes can be mainly classified into single cycle handling and dual cycle handling in marine container terminals. In the single cycle handling mode, the loading activities are handled after all unloading tasks have been finished. Dual cycle handling was first given the benefits described by Goodchild and Daganzo in 2006 [16]. This mode allows the crane to carry a container while moving from the apron to the ship (one move) immediately after moving a container from the ship to the apron, doubling the number of containers transported in one cycle (or two moves) [17]. To compare with single cycle handling, dual cycle handling decreases more empty movements of crane and observably reduces the ship turn-around time so as to increase the transshipment terminal productivity.

In this paper, our RMGC scheduling optimization is based on hybrid handling mode which mixes single cycle and dual cycle handling. After a VAC unloading operation, the next operation could be TUC unloading operation, VLC loading operation, TLC loading operation, or VAC unloading operation. All loading and unloading operations of one task are mixed. The next handling type of one operation (loading or unloading) is determined based on the demands of RMGC scheduling optimization in this paper.

### 3.4. Scheduling Objective

In this paper, we define a handling task as a loading-unloading operation of per-RMGC for a cluster which includes loading-unloading operations in rail handling track and truck operation lane in the fixed handling area.

The RMGC handling time *T* is composed by loading-unloading time *T*_*L*-*U*_ and idle load time *T*_*m*_ which is the moving time between two handling operations. As the handling operation positions are known, the *T*_*L*-*U*_ is a fixed value. Therefore, the *T*_*m*_ is the only determinant of handling time and is affected by handling sequence.

Based on the analysis above, in this paper, the objective of studying the RMGC scheduling problem is to determine the sequence of loading-unloading operations, whose idle load time of RMGC in handling task is minimized.

## 4. A Mathematical Formulation

In this section, a mathematical formulation for the RMGC scheduling problem in railway container terminals is proposed. The following six assumptions are introduced for the problem formulation.

1. Each vehicle and truck loading-unloading operation involves only one container once.
2. Handling locations of containers are assumed to be known and fixed before handling operations.
3. All handling operations in one task are nonpreemptive; that is, once an RMGC starts to do an operation, it must complete it without any pause or shift.
4. The containers in the model are assumed to be of the same size.
5. The containers are assumed to not be rehandled in the handling task.
6. The stop position of each vehicle on the rail handling track is in the same column of bay in the fixed handling area.

### 4.1. Notations and Variables

The following notations are used for a mathematical formulation:

 
*N* : the total number of handling tasks for per-RGMC in fixed handling area;
 
*i*, *j*: operations indices: operations are ordered in an increasing order and a handling task includes several operations;
 
*B*: the total number of bays in fixed handling area;
 
*L*: the total number of rows in fixed handling area (including 2 rail handling tracks and 1 truck operation lane);
 
*a*, *b*, *c*, *d*: the bay indices of operation positions: bays are ordered in an increasing order from left to right in the schematic representation of railway container terminal;
 
*k*, *l*, *e*, *m*: the row indices of operation positions: rows are ordered in an increasing order from rail handling track to truck operation lane in the schematic representation of railway container terminal;
 (*a*, *k*): the operation positions indices;
 
*d*_(*a*,*k*),(*b*,*l*)_: the moving distances of RMGC from (*a*, *k*) to (*b*, *l*);
 
*v*: the average moving speed of RMGC;
 
$\tilde{T}$: the set of tasks;
 
$\tilde{P}$: the set of operation positions;
 
*M*: a sufficiently large constant.

The decision variables are defined as follows:

 
*st*_(*a*,*k*),(*b*,*l*)_^*i*^: the start time of the *i* container handled from (*a*, *k*) to (*b*, *l*);
 
*ct*_(*a*,*k*),(*b*,*l*)_^*i*^: the finish time of the *i* container handled from (*a*, *k*) to (*b*, *l*);
 
*t*_(*c*,*e*),(*a*,*k*)_^*ji*^: the moving time of RMGC from (*c*, *e*) to (*a*, *k*) while the *i* container handled immediately begins after the *j* container handled has been finished;
 
*X*_(*c*,*e*),(*a*,*k*)_^*ji*^: 1, if the *i* container handled immediately begins after the *j* container handled has been finished, and 0, otherwise;
 
*c*_*i*_: 1, if the *i* container is the last container of handling task, and 0, otherwise;
 
*s*_*i*_: 1, if the *i* container is the first container of handling task, and 0, otherwise.

### 4.2. Objective Function

According to the problem description in Section 3, the objective function of RMGC scheduling optimization can be formulated as follows:

(1) $$\begin{array}{c} \text{Minimize}\overset{N}{\underset{j=1}{\sum }}\;\overset{N}{\underset{i=1}{\sum }}X_{\left(c,e),(a,k\right)}^{ji}t_{\left(c,e),(a,k\right)}^{ji}. \end{array}$$

The objective function of RMGC scheduling problem is to determine an optimization handling sequence in order to minimize the RMGC idle load time of handling task in the fixed handling area.

### 4.3. Constraints

The constraints of RMGC scheduling optimization are introduced as follows to ensure the practical feasibility of the solution.

(1) Handling time constraints,
  (2) $$\begin{array}{c} ct_{\left(a,k),(b,l\right)}^{i}-st_{\left(a,k\right),\left(b,l\right)}^{i}\leq \frac{d_{\left(a,k\right),\left(b,l\right)}}{v},\;i=1,2,\ldots ,n, \end{array}$$
  (3) $$\begin{array}{c} t_{\left(c,e),(a,k\right)}^{ji}=\frac{d_{\left(c,e),(a,k\right)}}{v},\;i,j=1,2,\ldots ,n, \end{array}$$
  (4) ct(d,m),(c,e)j+t(c,e),(a,k)ji−sta,k,b,li≤M1−Xc,e,a,kji,∀i,j∈T~,  ∀(a,k),(b,l),(c,e),(d,m)∈P~.

Equation (2) is the operation time constraint and ensures that one handling operation time should be less than or equal to the operation moving distances divided by average moving speed of RMGC. Equation (3) is the moving time constraint of sequential handling operations and indicates that the moving time between two sequential operations equals the moving distances between two operations divided by average moving speed of RMGC. Equation (4) is the time relationship constraint between sequential handling operations and indicates that the start time of subsequent operation cannot be earlier than the sum of preorder operation finish time and moving time between two operations.

 (2) Handling sequence constraints,
  (5) $$\begin{array}{c} \overset{N}{\underset{j=1}{\sum }}X_{\left(a,k),(b,l\right)}^{ji}\leq 1,\;\forall i\in \tilde{T},\;\forall \left(a,k\right),\left(b,l\right)\in \tilde{P}, \end{array}$$
  (6) $$\begin{array}{c} \overset{N}{\underset{i=1}{\sum }}X_{\left(a,k),(b,l\right)}^{ji}\leq 1,\;\forall j\in \tilde{T},\;\forall \left(a,k\right),\left(b,l\right)\in \tilde{P}, \end{array}$$
  (7) $$\begin{array}{c} \overset{N}{\underset{i=1}{\sum }}s_{i}=1, \end{array}$$
  (8) $$\begin{array}{c} \overset{N}{\underset{i=1}{\sum }}c_{i}=1. \end{array}$$

Equation (5) is the preorder operation constraint and indicates that each handling operation has at most one preorder operation. Equation (6) is the subsequent operation constraint and indicates that each handling operation has at most one subsequent operation. Equation (7) is the beginning operation constraint and ensures the handling task only has one beginning operation position in fixed handling block at a scheduling period. Equation (8) is the finished operation constraint and ensures one handling task only has one finished operation position in fixed handling block at a scheduling period.

## 5. An Ant Colony Optimization Algorithm for the Problem

The crane scheduling problem has proved to be NP-hard [5, 18]. So the formulation proposed above cannot be exactly solved in reasonable time. In this section, we propose an ant colony algorithm to obtain the approximate optimal solution of RMGC scheduling problem in railway container terminals.

Ant colony optimization (ACO) algorithm is a well-known metaheuristic approach, based on the behavior of ants seeking a path between their colony and a source of food. It is initially proposed by Marco Dorigo in 1992 in his Ph.D. thesis and has been successfully applied to solve several NP-hard optimization problems. Currently, ACO algorithms have been widely used in various fields of engineering applications like network, transportation, manufacturing, and so forth.

Main steps of the ACO algorithm implementation proposed in this paper are introduced in the following subsections.

(*1) Critical Parameters Setting.* ACO algorithms have some critical parameters that influence the performance dramatically, such as the heuristic coefficients *α*, *β* and pheromone hangover coefficient *ρ*. In this paper, the parameters values are determined by the simulation method.

 (*2) Transition Rule.* The transition direction of the ant *z*  (*z* = 1, 2,…, *m*) is determined by the operation sequence intensity in the ant moving process, and *p*_*ij*_^*z*^(*t*) is the transition probability of the ant *z* moving from operation *i* to operation *j* in period *t*, which is calculated by

(9) pijz(t) =τijtα·ηijtβ∑w⊂allowedzτiwtα·ηiwtβ,  j∈allowedz0,  otherwise,

where *τ*_*ij*_(*t*) is the operation sequence intensity between operation *i* to operation *j*, *η*_*ij*_(*t*) is the visibility of operation *i* to operation *j*, *η*_*ij*_(*t*) = 1/*d*_*ij*_. *d*_*ij*_ is the distance between operation *i* and operation *j*. allowed_*z*_ is the set of optional operations. The operation sequence intensity can be described as an adaptive memory and is regulated by the parameter *α*. The latter criteria can be described as a measure of desirability and are called visibility. It represents the heuristic function mentioned above and is regulated by the parameter *β*.

(*3) Pheromone Updating.* In order to avoid heuristic information covered by pheromone hangover, the pheromone need be updated when all ants accomplish one circulation. The pheromone of operation sequence in period *t* + *n* can be undated by

(10) $$\begin{array}{c} \tau_{ij}\left(t+n\right)=\left(1-\rho \right)\cdot \tau_{ij}\left(t\right)+\Delta \tau_{ij}\left(t\right),\\ \Delta \tau_{ij}\left(t\right)=\overset{m}{\underset{k=1}{\sum }}\Delta \tau_{ij}^{z}\left(t\right), \end{array}$$

where *ρ* (0 < *ρ* < 1) is the pheromone hangover coefficient. Δ*τ*_*ij*_(*t*) is the pheromone increment of operation sequence (*i*, *j*). Δ*τ*_*ij*_^*z*^(*t*) is the pheromone embedded in operation sequence (*i*, *j*) by the ant *z* in the circulation. If the ant *z* passes the (*i*, *j*) in this circulation, Δ*τ*_*ij*_^*z*^(*t*) = *Q*/*L*_*z*_. Otherwise, Δ*τ*_*ij*_^*z*^(*t*) = 0. *Q* is the pheromone amount released by the ant *z* in one circulation. *L*_*z*_ is the moving distance amount of the ant *z* in one circulation.

**Figure 3:**
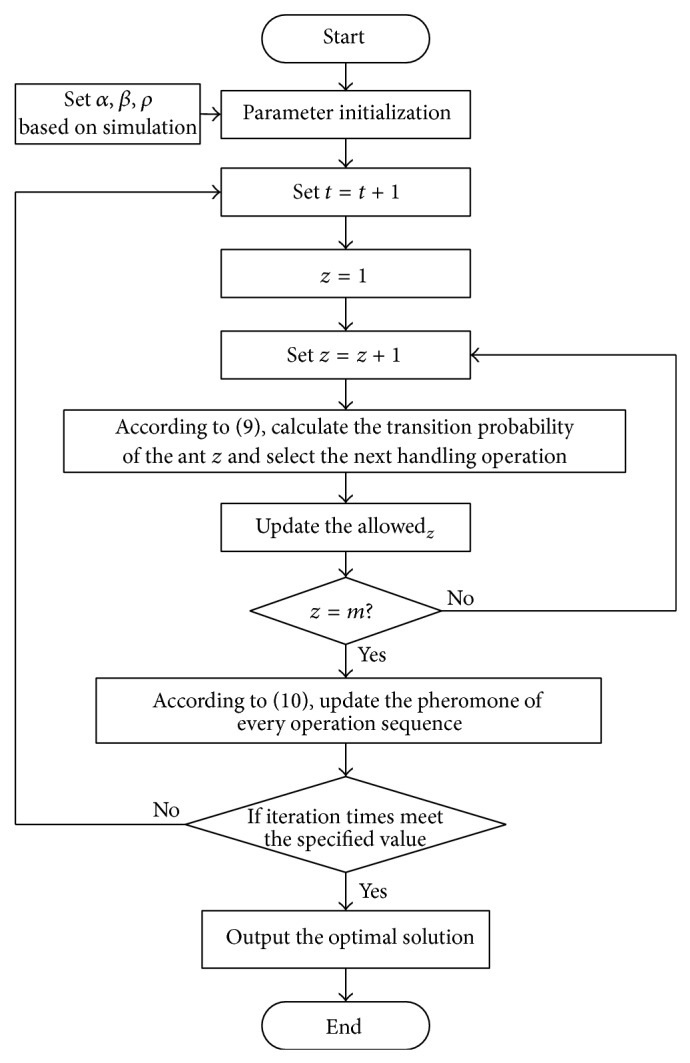
Flowchart of the ant colony optimization algorithm.

The flowchart of the ant colony optimization algorithm proposed in this paper is shown in Figure 3.

## 6. Computational Experiments

In this section, computational experiments are performed to illustrate the proposed model and algorithm for the RMGC scheduling problem in railway container terminals based on a specific railway container terminal in China. A comparison is made to assess the improvement between our approach (OA) and current approach (CA) used in railway container terminals. According to the current approach, firstly, VAC are orderly handled from left to right in the unloading rail track, then VLC are handled from left to right based on the loading sequence in the loading rail track, and truck loading-unloading containers are operated finally. Furthermore, to evaluate the practicability and effectiveness of OA, computational experiments in the different sizes of handling tasks are carried out. These numerical experiments are performed based on a personal computer with Intel Core (TM) 2.50 GHz processors and 4 GB RAM.

The parameters related to the specific railway container terminal are described as follows. The terminal has 2 rail handling tracks (with 120 operation positions each track), 1 truck operation lane, 2–4 RMGCs, 6 lanes, and 120 bays of main container yard. A handling task in the fixes area with sample size 65 is shown in Table 1.

According to the parameters values simulation, the parameters are set as follows: *α* = 5, *β* = 1, and *ρ* = 0.1. Experiments based on the computational sample in Table 1 are conducted for 50 independent runs. Then, a comparison between OA and CA is conducted to evaluate the performance of our approach for RMGC scheduling, which is shown in Table 2.

As observed in Table 2, the gap of idle load time of RMGC in the handling task between solutions obtained from the OA and CA is 56.8%, and the gap of total time of RMGC in the handling task between solutions obtained from the OA and CA is 23.2%. All the computational time of these experiments is short. Based on the gaps mentioned above, it is clear that near optimal solutions obtained from our approach prominently reduce the idle load time and the total time of handling task. The reductions of idle load time of RMGC can directly improve efficiency of handling operations and indirectly reduce the waiting time of container trains and trucks.

To evaluate the effectiveness and reliability of the proposed RMGC scheduling approach in this paper, several computational experiments in different sample sizes are carried out. For each sample size, the experiments are conducted for 50 independent runs to evaluate the performance of our approach for different sample sizes. The computational result is shown in Table 3.

As observed in Table 3, the computational time of different sample sizes is in the acceptable time range, and the gaps of idle load time of RMGC in handling task between solutions obtained from the OA and CA are more than 40%. The performance of our approach is satisfactory in solving different size instances. The computational experiment results indicate that our approach is efficient to solve RMGC scheduling problem and can markedly reduce the RMGC idle load time and can shorten the total time of the handling task. The RMGC scheduling optimization is significant for the operation and organization of railway container terminals.

## 7. Conclusion

In this paper, we considered the RMGC scheduling problem in railway container terminals based on hybrid handling mode. The main contributions of this paper are concluded as follows. Firstly, we analyze the handling area, objects, mode, and scheduling objective of RMGC scheduling problem in railway container terminals. Then, according to the problem description, an RMGC scheduling optimization model was proposed, whose objective is to minimize the RMGC idle load time of handling task. An ant colony optimization algorithm was designed to obtain the optimization handling sequence. Finally, computational experiments on a specific railway container terminal in China showed that the method in this paper is effective in solving RMGC scheduling problem in railway container terminals and has a good performance for different size instances. In future, considering the multi-RMGCs scheduling problem with intercrane interference in railway container terminal is a possibility for further research.

## Figures and Tables

**Table 1:** Handling task under sample size 65.

Number	Type	Start position	Final position	Number	Type	Start position	Final position
1	VAC_1_	UL1	(2, 2)	2	VAC_1_	UL2	(1, 1)
3	VAC_1_	UL3	(1, 2)	4	VAC_1_	UL4	(1, 5)
5	VAC_1_	UL5	(2, 3)	6	VAC_2_	UL6	T1
7	VAC_1_	UL7	(2, 9)	8	VAC_1_	UL8	(1, 6)
9	VAC_1_	UL9	(2, 7)	10	VAC_1_	UL10	(1, 9)
11	VAC_1_	UL11	(1, 10)	12	VAC_1_	UL12	(2, 13)
13	VAC_1_	UL13	(2, 11)	14	VAC_1_	UL14	(1, 15)
15	VAC_1_	UL15	(1, 14)	16	VAC_1_	UL16	(3, 17)
17	VAC_1_	UL17	(1, 18)	18	VAC_2_	UL18	T17
19	VAC_1_	UL19	(1, 20)	20	VAC_1_	UL20	(2, 19)
21	VAC_2_	UL21	T23	22	VAC_1_	UL22	(1, 24)
23	VAC_1_	UL23	(1, 21)	24	VAC_1_	UL24	(1, 25)
25	VAC_1_	UL25	(3, 24)	26	VAC_1_	UL26	(2, 28)
27	VAC_1_	UL27	(1, 26)	28	VAC_1_	UL28	(1, 29)
29	VAC_1_	UL29	(1, 28)	30	VAC_1_	UL30	(2, 29)
31	VLC	(5, 2)	L1	32	VLC	(4, 1)	L2
33	VLC	(6, 1)	L3	34	VLC	(6, 6)	L4
35	VLC	(6, 3)	L5	36	VLC	(5, 8)	L6
37	VLC	(6, 5)	L7	38	TUC_2_	T7	L8
39	VLC	(6, 10)	L9	40	VLC	(6, 8)	L10
41	VLC	(5, 12)	L11	42	VLC	(6, 11)	L12
43	VLC	(5, 11)	L13	44	VLC	(6, 17)	L14
45	VLC	(6, 14)	L15	46	VLC	(4, 15)	L16
47	VLC	(5, 19)	L17	48	VLC	(5, 17)	L18
49	VLC	(6, 18)	L19	50	VLC	(6, 22)	L20
51	VLC	(6, 20)	L21	52	TUC_2_	T20	L22
53	VLC	(5, 20)	L23	54	VLC	(6, 25)	L24
55	VLC	(5, 24)	L25	56	VLC	(6, 29)	L26
57	VLC	(5, 28)	L27	58	VLC	(6, 27)	L28
59	VLC	(5, 30)	L29	60	VLC	(6, 28)	L30
61	TUC_1_	T3	(5, 1)	62	TUC_1_	T6	(3, 7)
63	TUC_1_	T10	(1, 12)	64	TUC_1_	T16	(1, 17)
65	TUC_1_	T27	(4, 29)				

Notes: UL^*^ denotes the operation position indices in rail unloading track; L^*^ denotes the operation position indices in rail loading track; T^*^ denotes the operation position indices in truck operation lane; (*a*, *k*) denotes the operation positions indices in container yard.

**Table 2:** Comparison between OA and CA in sample size 65.

OA							CA		GAP_1_	GAP_2_
Idle load time (min)			Total Time (min)	CPU (s)			Idle load time (min)	Total time (min)		
Max	Min	Avg.		Max	Min	Avg.				
26.1	23.8	24.5	106.7	55.6	55.2	55.4	56.7	138.96	56.8%	23.2%

Notes: GAP_1_ = (idle load time of RMGC obtained from CA − average idle load time of RMGC obtained from OA) ∗ 100/idle load time of RMGC obtained from CA; GAP_2_ = (total time of RMGC obtained from CA − average total time of RMGC obtained from OA) ∗ 100/total time of RMGC obtained from CA.

**Table 3:** Performance of OA for different sample sizes.

Sample size	CPU (s)			GAP_1_	GAP_2_
	Max	Min	Avg.		
70	62.7	60.3	61.4	54.2%	22.7%
100	148.9	145.7	146.2	49.2%	18.7%
130	273.6	245.3	257.9	45.2%	13.9%
